# Congenital Cystic Adenomatoid Malformation of the Lung Tipe II: Three Cases Report

**Published:** 2019-01-12

**Authors:** A Garzi, U Ferrentino, G Ardimento, S Brongo, M.S Rubino, E Calabrò, E Clemente, R.M Di Crescenzo

**Affiliations:** 1 Division *of Pediatric* M.I.S. and Robotic Surgery University of Salerno; 2 Division of Pediatric Surgery University of Salerno; 3 Division of Plastic Surgery University of Salerno; 4 Department of Advanced Biomedical Sciences, Pathology Unit, University of Naples Federico II

**Keywords:** Congenital cystic adenomatoid malformation (CCAM), Congenital pulmonary airway malformation (CPAM), Bronchopulmonary sequestration, Congenital bronchopulmonary malformation, Sparing surgery

## Abstract

Congenital cystic adenomatoid malformation (CCAM) is a rare congenital lung lesion. It may appear since birth (30–35%) with difficulty breathing or may have a late onset (60–65%) with recurring pulmonary infections or growth failure; in a small percentage of cases, the lesion can be completely asymptomatic.

Fetal or post-natal surgery can be used as surgical treatment of these lesions. Postnatal surgery consists of a lobectomy, bilobectomy or pneumonectomy, based on the size of the lesion. The best age to undergo this surgery is around 2 years, but only if the injury is stable and the child has no complications.

The study describes three cases of CCAM, observed at the Pediatric Surgery Section of the University of Siena. We analyzed those 3 cases whose approach was defined by the onset of symptoms, age and clinical condition of patients. In the first case the surgery was performed a few hours after birth due to the worsening of the clinical conditions; in the other two cases it was delayed because the patients were asymptomatic.

The purpose of this study is to review the management of patients with CCAM in relation to clinical onset and the type of injury.

## I. INTRODUCTION

Congenital cystic adenomatoid malformation (CCAM) is a rare hamartomatous lesion of the lung, characterized by a cystic mass of dysplastic pulmonary tissue with proliferation of bronchial structures to the prejudice of alveoli. The lesion can manifest itself with the development of respiratory distress, sometimes life threatening, or with recurrent pulmonary infections.

The malformation is usually confined to a single lobe, without right or left predominance, and is rarely bilateral. The incidence of CCAM varies from 1:25.000 to 1:35.000, even if a better knowledge of this affection makes the diagnosis more frequent (1, 2).

In 1949 Chin and Tang first reported a case of CCAM (3); it was later studied thoroughly by Kwitten and Reiner in 1962 and it was classified by Stocker in 1977 (4).

The Authors describe three cases of type II CCAM, observed at the Section of Pediatric Surgery of the University of Siena. The purpose of this study is to review the management in relation to the clinical onset and the type of the lesion.

### Case 1-

**Fig. 1:**
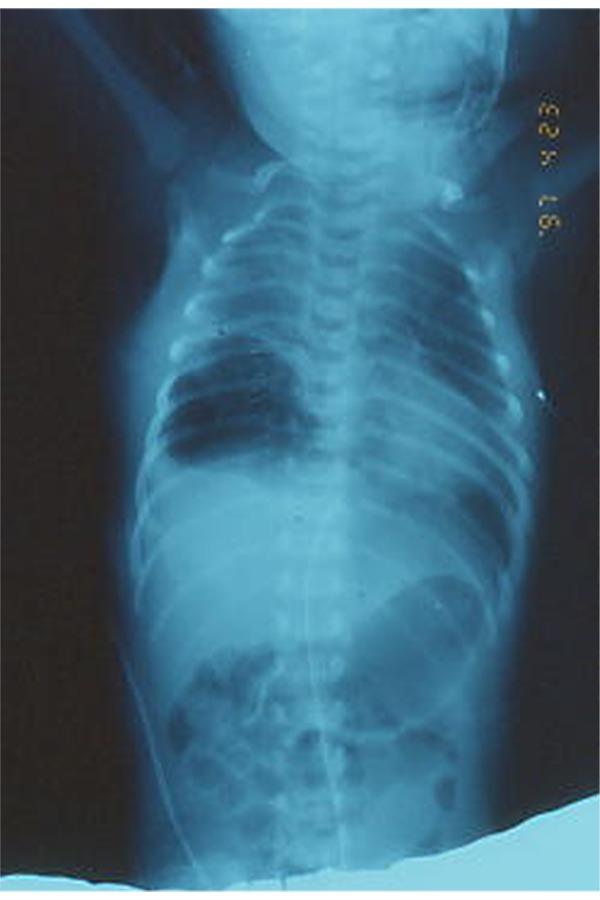
Chest radiograph: multicystic lesion of the right lower lobe with mediastinal left shift.

**Fig. 2:**
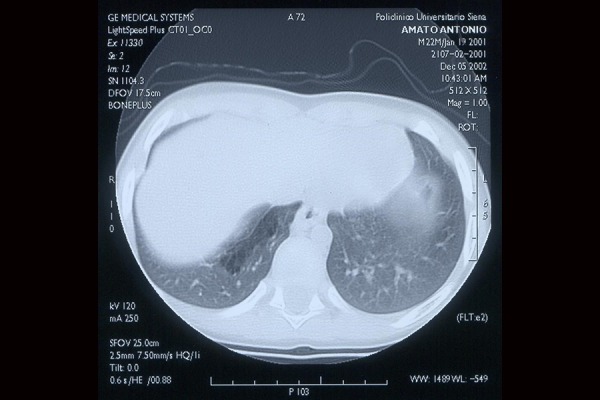
Computed Tomography: multicystic lesion of the right lower lobe.

F.S., female born at the 39th weeks of gestation, by emergency caesarean section due to fetal distress (late decelerations detected upon fetal heart-rate monitoring). Repeated routine antenatal ultrasound (US) examinations were normal. Birth weight was 2.620 g and Apgar scores were respectively 6 and 10 at 1 and 5-min. A diagnosis of esophageal atresia was suspected since it was not possible to pass a nasogastric tube into the stomach. On admission to the neonatal intensive care unit the general condition was satisfactory, and the baby did not require mechanical ventilation. The initial diagnosis was confirmed by a plain radiographic examination; moreover, the contemporary initial chest roentgenograms showed a patchy, nonhomogeneous increase in density in the right lower lobe, which was interpreted as an aspiration pneumonitis due to the oesophageal atresia. Further investigations excluded any associated anomalies. Fourteen hours after birth a surgical operation was carried out dividing and closing the tracheoesophageal fistula and repairing the oesophageal atresia. After the operation the infant was placed on mechanical ventilation and in total parenteral nutrition. Two days after the operation, a multicystic lesion, involving the right lower lobe and resulting in heart and mediastinal left shift, became evident at the chest radiographs and computed tomography (Fig. 1 and 2).

**Fig. 3:**
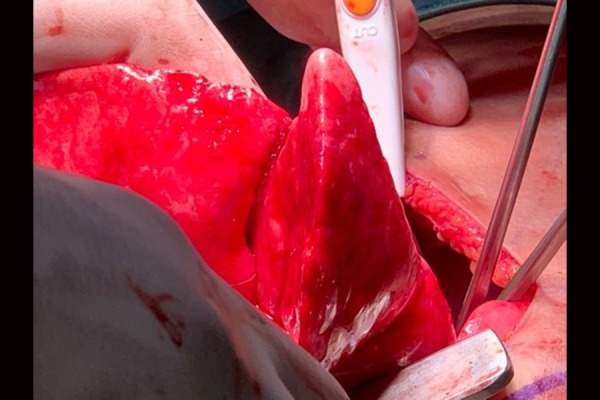
Macroscopic imagine of the lesion.

At 79 hours of life, a sudden change in vital signs, with an increase in oxygen requirements, tachypnea, cyanosis, a decrease in breath sounds up to the left hemitorax, and subcutaneous emphysema took place. A chest roentgenogram showed a small right pneumothorax, a left tensive pneumothorax, a pneumomediastinum, and a pneumoperitoneum. After a needle thoracentis, a second thoracotomy was undertaken. The lesion appeared to be multilocular, confined to the right lower lobe, and in communicating with airways. A segmental resection of the lesion was performed (Fig. 3).

The hystologic features were compatible with a diagnosis of type II CCAM. The postoperative course was uneventful, and the infant was discharged at 1 month of age. Last follow-up, at 6 years of age, showed normal ventilation and oesophageal transit.

### Case 2 -

A.A. male born at the 37th week of gestation. During 22 weeks of gestation an antenatal ultrasound examination showed some “cystic lesions, 12 mm in diameter, that took upthe whole right lung and caused mediastinal shift”; this lesion was found also in other two ultrasound examinations undertaken at the 25th and 27th gestation weeks. The latest ultrasound examinations, at the 33rd and 36th gestation weeks, showed a “reduction of iperechogenicity in pulmonar right lower lobe; the cystic lesions described before was not found”.

Birth weight was 2.850 g, Apgar score was 3 at 1-min. The infant immediately required mechanical ventilation. Amniotic fluid was sucked from portex, stomach and nose. Two days after the delivery the infant showed a good metabolic equilibrium, so he was extubated.

Eight days after birth a chest tomography showed a “reduced expansion of the lower right lobe, with some hyperdiaphan cystic areas, smaller than 1 cm in diameter”. Further examinations excluded associated anomalies and the child was discharged at 15 days of age because of normal growth, good general condition and normal clinical objectivity.

Further tomography examinations, at 2, 6 and 15 months of age, showed reduction of the cysts in the upper segment of the lower right lobe. The last examination, at 23 months of age, showed an increase in number and diameter of the cysts in the apical segment (Picture 4).

**Fig. 4:**
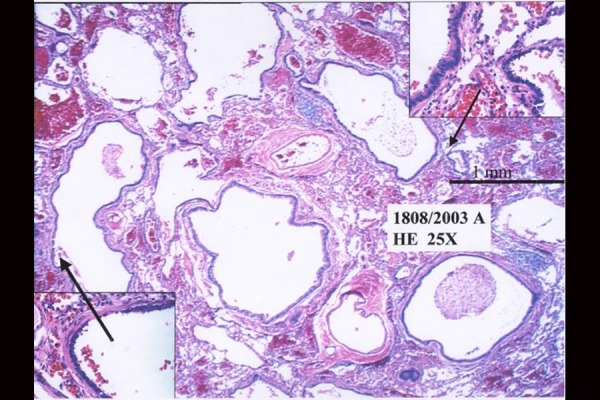
Hystological report: microscopic images.

**Fig. 5:**
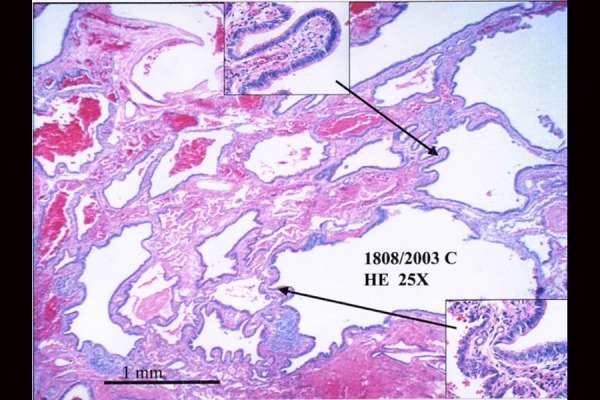
Histologicalreport: microscopic images

A lower right lobectomy was decided to avoid complications, at 24 months of age. Hystological report confirmed diagnosis of type II CCAM ( Fig. 4–5).

The day after surgery the child was hemodinamically stable, apyretic and well reactive, for these reasons he was extubated. Three hours later respiratory difficulties arose, and the child was immediately reintubated. Performing a bronchoscopy few secretions at the bronchial right base were found.

Four days after surgery, the child was extubated again but because of broncho spasms and laryngeal oedema, arisen 20 minutes later, re-intubation was tried. Due to the impossibility to perform this operation a tracheotomy was necessary, then removed 6 days later.A tracheotomy was necessary due to the impossibility to perform this operation,then removed 6 days later.

Chest radiographs showed a progressive expansion of residual lung tissue and a very slight pneumothorax in the post-operative period.

General condition was good, so the child was discharged 20 days after surgery.

At the follow-up, at 1 and 3 months after surgery, the child had a good general condition and the chest x-ray showed a complete expansion of the lung and reabsorption of the pneumothorax.

### Case 3 -

G.S., female, born at the 38th weeks of gestation; repeated routine antenatal ultrasound (US) examinations were normal. Birth weight was 2.960 g and Apgar scores wererespectively8 and 10 at 1 and 5-min. On admission to the neonatal intensive care unit the general condition was satisfactory, and the baby did not require mechanical ventilation. The initial diagnosis was confirmed by a plain radiographic examination.Eight days after birth a chest tomography showed a “reduced expansion of the lower left lobe, with some hyperdiaphan cystic areas, smaller than 2 cm in diameter”. Further examinations excluded associated anomalies and the child was discharged at 10 days of age because of normal growth, good general condition and normal clinical objectivity.

**Fig. 6:**
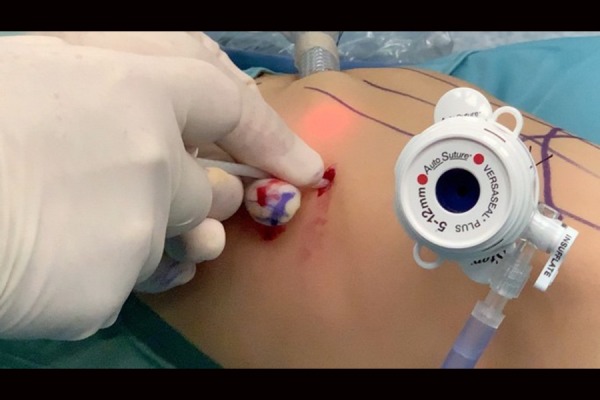
Thoracoscopic approach.

The lesion appeared to be multilocular, confined to the left lower lobe and lingula, and in communicating with airways. A resection of lower lobe and lingual was performed at 3 years and 8 months of age. It was used a thoracoscopic approach with subsequent thoracotomy conversion (Fig. 6).

The histologic features were compatible with a diagnosis of type II CCAM. The postoperative course was uneventful and the infant was discharged third days later. Last follow-up showed normal ventilation and normal lung expansion.

## II. DISCUSSION

CCAM was first classified by Stocker in 1977 according to the number and the size of cysts, and to histology (4, 5, 6).

CCAM type I (70%) includes single or multiple cysts, more than 2 cm in diameter and lined by ciliated pseudostratified columnar epithelium. Structures resembling normal alveoli lay between the cysts.

Type II (20%) consists of multiple small cysts less than 1 cm in diameter that are lined by ciliated cubical to columnar epithelium and creating compression of the remaining lung tissue.

Type III (10%) includes large and non cystic malformation that usually produces mediastinal shift. Few patients survive at the last one or its associated anomalies.

Recently type IV has been described, with an acinar-alveolar type of epithelium like type I pneumocytes (7).

CCAM results from an anomaly of lung development between the 5th and 6th weeks of gestation, even if etiopathogenesis remains unknown (8, 9, 10, 11).

CCAM may be associated with other congenital anomalies such as: extralobular sequestration, congenital diaphragmatic hernia, lung hypoplasia, cardiovascular malformations, hydrocephalus, bones malformations, oesophageal atresia, bowel atresia, bilateral renal agenesia/disgenesia, Pierre-Robin Syndrome, Prune-Belly Syndrome (12, 13, 4, 7).

It is interesting to note that recent observations have suggested that tracheoesophageal malformations and congenital pulmonary defects may share a common embryological origin, with an incidence rate of 2,1%. This hypothesis may explain, from a pathogenic point of view, the association between type II CCAM and oesophageal atresia observed in the first case report (13, 14, 15).

From a clinical point of view, CCAM may present at birth (30–35%) with respiratory distress; another group of patients have a late onset (60–65%), with recurrent pulmonary infections or growth retard; the lesion can sometimes be completely asymptomatic and may only come to attention with a fortuitous X-rays.

CCAM can be diagnosed by antenatal ultrasound examination as early as the 12th week of intrauterine life (16, 17, 18). Early diagnosis is very important to plan delivery in third level specialist centres, to reduce risks due to respiratory distress, and to deal with associated malformations. In the first case we studied we did not make prenatal diagnosis, so that clinical picture developed after iperinsufflation.

Differential diagnosis includes all lung malformations (pulmonary sequestration, lobar emphysema, bronchogenic cysts), all the acquired inflammatory diseases, staphylococcus pneumopaty, and finally traumatic, accidental or iatrogenic emphysema and atelectasis.

The evolution of the lesion may be various. Spontaneous prenatal reduction or resolution of CCAM is well reported in the Literature, as well as postnatal spontaneous and complete resolution (19, 20). In the second child a postnatal reduction of the lesion has been observed.

Prognosis of neonatal CCAM is related to early diagnosis and relative treatment, so related to prognosis of pulmonary resection too.

Surgical failures, especially in infants with respiratory distress, is generally due to hypoplasy of controlateral lung, as a result of foetal compression operated by the malformed lung (21, 22).

CCAM is included among the indication of foetal surgery. This approach requires a complete and careful assessment of mother and foetus’ conditions. Selection criteria include hydropic foetus between 24 and 32 weeks gestation (1, 23, 24).

Postnatal surgery consists in lobectomy or bilobectomy or pneumonectomy if the lesion is bigger. A segmentary resection of lung tissue is not well accepted even when lesions do not completely take up the lobe because cysts isolation and edges identification are very difficult. However, as we saw in the first case, if the lesion is easy to take out from the lung tissue, the only segmentary resection may be performed.

In the last decade, thanks to technical improvement and better instruments, thoracoscopic approach have taken a very important role in childhood surgery. In fact, it is associated with excellent diagnostic and therapeutic results with low mortality rate and high benefits. Selection criteria are yet disputed; the efficacy of videoassisted procedure should be judged case by case (25).

Most Authors agree that asymptomatic patients should be monitored. Infact, if lesions do not show progressive growth and children have not recurrent respiratory infections, it is better to wait until 2–3 years of age to perform surgery. The best age for surgery is nearby 2 years: children stand better for thoracotomy, have less complications and a better growth ofresidual lung tissue. Our approach agrees with the strategies described before and confirms its validity.

## III. CONCLUSIONS

CCAM is a rare congenital lesion of the lung; its incidence increases due to the large improvement of antenatal ultrasound examination.

We reported on three cases defining therapeutic approaches on the grounds of onset, age and clinical condition of patients. In fact, in the first case report surgery was performed a few hours after delivery due to worsening of clinical condition; in the other case report surgery was delayed because the child was asymptomatic.

Besides, prenatal diagnosis is very important to reduce perinatal risks and to better deal with complication that may arise from this malformation.

Finally, reviewing the Literature we noted an agreed attitude to perform surgery between 12 and 24 months of age in all asymptomatic patients.

In fact, this range of age implies better tolerability and better results both short and long-term.
